# Inter‐operator and inter‐device reproducibility of shear wave elastography in healthy muscle tissues

**DOI:** 10.1002/acm2.13717

**Published:** 2022-07-06

**Authors:** Anna S. Vuorenmaa, Eetu M. K. Siitama, Katri S. Mäkelä

**Affiliations:** ^1^ Department of Medical Physics Medical Imaging Center Tampere University Hospital Pirkanmaa Hospital District Tampere Finland; ^2^ Department of Clinical Neurophysiology Medical Imaging Center and Hospital Pharmacy Tampere University Hospital Pirkanmaa Hospital District Tampere Finland

**Keywords:** interobserver reliability, neuromuscular ultrasound, reproducibility, shear wave elastography

## Abstract

**Purpose:**

The study aimed to assess whether the more limiting factor in reproducibility of shear wave elastography (SWE) would be the operator dependency or the incompatibility of different ultrasound (US) devices. The interrater agreement with less experienced operators was studied.

**Methods:**

A total of 24 healthy volunteers participated in the study (18 females, 6 males; range of age 27–55 years). SWE of biceps brachii (BB) and tibialis anterior (TA) muscles was performed on both sides from all participants in both longitudinal and transverse orientation of the transducer in respect to muscle fibers. Two operators repeated the SWE with two different US devices from different manufacturers (scanners 1 and 2).

**Results:**

Intraclass correlation coefficient between the two operators was 0.91 (CI 0.88–0.93) for scanner 1 and 0.81 (CI 0.74–0.86) for scanner 2, respectively. Instead, there were significant differences in the SWE measurements between the two scanners, emphasizing in transverse orientation of the transducer. In the transverse transducer orientation, the mean shear wave velocity (SWV) in TA was 1.45 m/s (standard deviation [SD] ± 0.35 m/s) with scanner 1 and 2.35 m/s (SD ± 0.83 m/s) with scanner 2 (*p* < 0.001). In BB, the mean transverse SWV was 1.49 m/s (SD ± 0.35 m/s) with scanner 1 and 2.29 m/s (SD ± 0.63 m/s) with scanner 2 (*p* < 0.001). In longitudinal transducer orientation, the mean SWV in TA was 3.00 m/s (SD ± 0.73 m/s) with scanner 1 and 3.26 m/s (SD ± 0.42 m/s) with scanner 2 (*p* = 0.050). In BB, the mean longitudinal SWV was 3.60 m/s (SD ± 0.77 m/s) with scanner 1 and 3.96 m/s (SD ± 0.62 m/s) with scanner 2 (*p* = 0.019). The presented mean values were obtained by operator 1, there were no significant differences in the SWE measurements performed by the two operators.

**Conclusion:**

The results implicate that the reproducibility of the SWE measurements depends rather on the used US device than on the operator. It is recommendable that clinics collect reference values with their own US device and consider threshold values presented in previous studies only directional.

## 1 INTRODUCTION

Shear wave elastography (SWE) is an ultrasound (US)‐based uprising form of the quantitative assessment of soft tissue physiology and pathology. SWE provides information on tissue stiffness by measuring the velocity of the propagating shear waves within the tissue. The shear wave technique is based on the speed of the shear wave $v_{s}$ (m/s), which is controlled by shear modulus *G* (Pa) and is related to focal tissue density. The shear force is generated using acoustic waves that propagate perpendicularly to the primary US wave at a lower velocity (typically 1–10 m/s) and lower frequency (typical range 10–500 Hz). The shear modulus describes the ability of a material to resist the force. The amount of shear is represented by the angle *θ*. The shear modulus *G* is represented in the following equation:

(1) $$G\;=\;\frac{\frac{F}{A}}{\tan\left(\theta \right)}$$

In SWE, the force, *F* (N), presses through an area *A* (m^2^). When the substance is compressed, it oscillates at the transmitted frequency. B‐mode follows the change in the speckle pattern, from which the propagation velocity can be deduced.

The following equation shows the speed of shear wave $v_{s}\;$ is controlled by shear modulus *G* and is related to tissue local tissue density *ρ*:

(2) $$\;v_{s\;}=\sqrt{\frac{G}{\rho }}\;$$

There are different algorithms to calculate the velocity from change in the speckle artifact, which are partially dependent on the manufacturer of the US device.^1^, ^2^

So far, SWE has been shown to be a reliable tool in diagnostics of liver fibrosis, breast cancer, and thyroid cancer.^3^ However, lately SWE has been introduced to have a promising role also in the diagnostics, determination of disease severity, and follow‐up in other tissues and pathologies, such as the musculoskeletal system and nerve entrapment neuropathies.^4^, ^5^, ^6^, ^7^, ^8^ SWE studies involving muscle disorders have focused mainly on myopathies, showing some incoherent study results.^4^, ^9^, ^10^, ^11^

As a relatively new diagnostic tool, SWE is still an evolving application and has some limitations that can also be considered potential biasing factors. The main well‐known limitations of SWE are the operator dependency and the incompatibility of the SWE applications of different US device manufacturers.^7^ SWE systems have mainly been studied previously using liver tissue‐mimicking phantoms.^12^ However, the adaptability of the phantom studies to clinical practice can be questioned, especially concerning anisotropic tissues. In anisotropic tissues, such as the muscle tissue, the propagation velocity is dependent on the position of the transducer in respect of the muscle fiber direction.^7^ Determination of the optimal angle may cause challenges, which potentially emphasizes the operator dependency of SWE in muscles, compared to isotropic tissues. The capability to produce reliable SWE measurements has been proposed to need expertise and years of user experience.^13^ Regarding the increasing interest in SWE in scientific research and clinical use, it is important to survey how these limitations impact the reproducibility of the SWE measurements. This reflects the comparability of the research articles as well as the repeatability of a clinical examination.

The aims of the study were to compare SWE measurements acquired by two different US devices of different manufacturers and to assess the interrater agreement of two operators with both devices. The study aimed to determine whether the more limiting factor in reproducibility of SWE would be the operator dependency or the proposed incompatibility of the different US devices. An interesting question was also the interrater agreement of less experienced operators. The repeatability of the SWE between days was also tested. The study focused to test the reproducibility of SWE in a clinical point of view, particularly in muscle tissue. Thus, the study was performed with healthy volunteers. The depth of the studied object in SWE seems to influence the reliability of the measurements, increasing the hazard of measure error, artifacts, and attenuation effect.^3^, ^6^ Biceps brachii (BB) and tibialis anterior (TA) muscles are relatively superficial with a simple anatomy and were therefore chosen for the studied muscles.

## 2 METHODS

In total, 24 healthy volunteers participated in the study. Of the 24 participants, 18 were women and 6 were men. The age ranged between 27 and 55 years (mean age 38 years, standard deviation [SD] ± 8.2 years). Overall, 2 of the participants were left‐handed and 22 right‐handed. All participants were hospital professionals and employees. All participants gave their written consent, and the board of ethics of the local health care district approved the study.

The participants considered their mobility normal and had no symptoms of any kind in their lower or upper limbs. In addition, conditions and related symptoms that may affect the tissue composition of the studied muscles, such as radiculopathy, focal peripheral neuropathy, or myopathy were asked specifically. One volunteer reported a diagnosed L5 radiculopathy and was therefore excluded from the study. SWE measurements were performed on both muscles and both sides on all participants, resulting in 48 studied BB and TA muscles in total.

**FIGURE 1:**
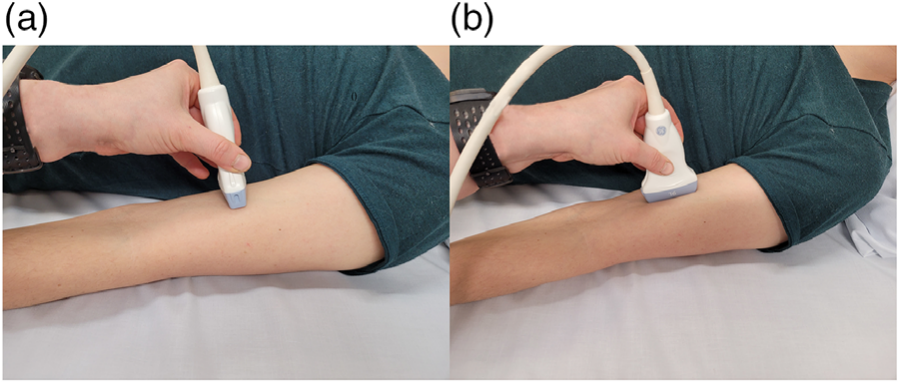
The figure demonstrates the transducer orientations on the biceps brachii (BB) muscle: (a) transverse transducer orientation; (b) longitudinal transducer orientation.

The participants were asked to relax in a supine position on an examination table with arms fully extended and palms facing upward. Coupling gel was applied on the skin surface, and the transducer was lightly placed on the skin, with caution not to apply any pressure to the muscle by pressing the transducer. Even slight pressure of the transducer has been shown to lead to incorrect results.^14^ The transducer was placed approximately in the middle in the length and width of the muscle. The transducer axis was determined by the orientation of the muscle fibers in the B‐mode image. Propagation velocity of the shear waves is dependent on the transducer orientation and is often studied in different dimensions.^15^ Thus, the muscles were studied in both transverse and longitudinal planes. The transducer orientations are demonstrated in Figure 1. Region‐of‐interest (ROI) diameter was 3 mm with both US devices, and it was adjusted approximately to the middle of the muscle bulk in depth. The most appropriate stiffness unit for muscle is the shear wave velocity (SWV).^13^ Three consecutive SWV measurements were taken, and the mean of the measurements was calculated and documented. The order of the operators to perform the measurements was randomized. The second operator to perform the measurements was allowed to see the probe placement of the first operator. However, the operators were blinded to the SWV values acquired by each other, and the values were documented by a third person.

The measurements were performed with two US devices of two different manufacturers. The US devices were GE LOGIQ S8 XDclear 2.0, with a linear transducer 9L‐D, frequency range 2–8 MHz (referred to in future as scanner 1), and Canon Aplio i800 with a linear transducer PLT‐1005BT, frequency range 4–14 MHz (referred to in future as scanner 2). The used application was 2D SWE that simultaneously transmits beams and displays a color image representing the tissue stiffness. In the 2D SWE technique, the elastogram and the B‐mode image are overlaid, and the user can adjust the position of the ROI. The manufacturers SWE default protocol was used in measurements.

To estimate the interrater agreement, two different healthcare professionals studied all muscles with both US devices. One of the operators was a resident medical physicist (referred to in future as operator 1) and the other a medical doctor specializing in clinical neurophysiology (referred to in future as operator 2). The operators did not have any previous user experience on SWE; however, both had an experience of ∼2 years of conventional B‐mode imaging. The sales directors of the respective scanners trained both operators for the SWE applications. The measurements of the second operator were done immediately after the first.

To study the steadiness of the SWE in a period of time, repeated SWE measurements were performed on one of the volunteers in the morning and afternoon on 4 days over a 1‐week period. In this setting, the studied muscle was the right BB, and the measurements were done with scanner 2. The methods of performing the SWE measurements were the same as previously described.

The statistical analyses were done using the SPSS software (SPSS versions 24 and 25, SPSS, Chicago, IL). Significances of the differences of SWV values between two groups were tested using the Mann–Whitney test. A *p*‐value <0.05 was considered significant. The interobserver reliability was tested using the intraclass correlation coefficient (ICC). Thresholds for ICC interpretation were: <0.2 poor agreement, 0.21–0.4 fair agreement, 0.41–0.6 moderate agreement, 0.61–0.8 good agreement, and >0.8 very good agreement.^16^

## 3 RESULTS

The effect of the operator dependency on the reproducibility of the SWE was studied by the interobserver reliability and tested with the ICC. The ICC between the two operators was tested separately with both scanners. ICC for average measurement was 0.91 (CI 0.88–0.93, standard error for measurement [SEM] 0.63) with scanner 1 and 0.81 (CI 0.74–0.86, SEM 0.76) with scanner 2. Minimal detectable change (MDC) was defined as three times the SD of the difference between operators’ SWV measurements. The MDC values are presented in Tables 1 and 2.

**TABLE 1:** The shear wave velocity (SWV, m/s) values measured in the transverse orientation

	Scanner 1					Scanner 2					
	Mean	Minimum	Maximum	SD	MDC	Mean	Minimum	Maximum	SD	MDC	*p*‐Value*
Operator 1, TA	1.45	0.90	2.37	0.35	±0.96	2.35	1.16	4.15	0.83	±3.27	<0.001
Operator 2, TA	1.37	0.82	2.55	0.32		2.46	1.14	4.90	1.10		<0.001
Operator 1, BB	1.49	0.90	2.39	0.35	±1.23	2.29	1.04	4.25	0.63	±2.16	<0.001
Operator 2, BB	1.58	1.03	2.65	0.30		2.33	1.40	3.87	0.64		<0.001

Abbreviations: BB, biceps brachii; MDC, minimal detectable change, defined as three times the standard deviation of the difference between operators’ SWV measurements; SD, standard deviation; SWV, shear wave velocity; TA, tibialis anterior.

*The *p*‐value for the difference of the mean SWV between the two scanners, according to the operator and the studied muscle.

**TABLE 2:** The shear wave velocity (SWV, m/s) values measured in the longitudinal orientation

	Scanner 1					Scanner 2					
	Mean	Minimum	Maximum	SD	MDC	Mean	Minimum	Maximum	SD	MDC	*p*‐Value*
Operator 1, TA	3.00	1.73	4.72	0.73	±2.31	3.26	1.88	4.26	0.42	±1.29	0.050
Operator 2, TA	2.99	1.76	4.46	0.66		3.25	2.06	4.18	0.43		0.044
Operator 1, BB	3.60	1.18	4.91	0.77	±2.46	3.96	2.35	5.50	0.62	±1.89	0.019
Operator 2, BB	3.63	2.32	4.91	0.73		3.86	2.75	5.05	0.57		0.109

Abbreviations: BB, biceps brachii; MDC, minimal detectable change, defined as three times the standard deviation of the difference between operators’ SWV measurements; SD, standard deviation; SWV, shear wave velocity; TA, tibialis anterior.

*The *p*‐value for the difference of the mean SWV between the two scanners, according to the operator and the studied muscle.

**FIGURE 2:**
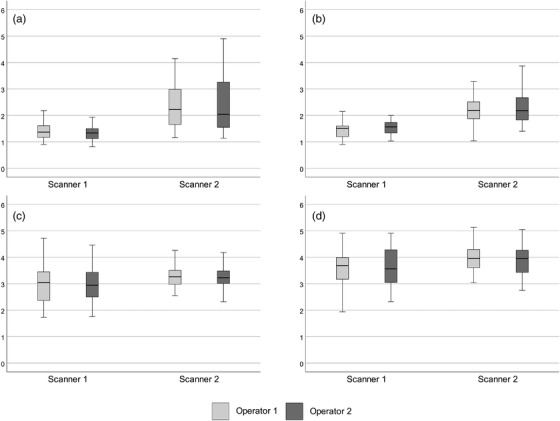
Boxplots showing both the difference in the shear wave velocity (SWV) values of the two ultrasound devices and the codirectional SWV measurements of the two operators. The *y*‐axis represents the SWV in m/s. The box indicates the first and third quartiles, the thick line within the box represents the median, and the bars represent the minimum and maximum values of SWV: (a) tibialis anterior (TA) muscle in transverse orientation; (b) biceps brachii (BB) muscles in transverse orientation; (c) TA in longitudinal orientation; and (d) BB in longitudinal orientation of the transducer.

When the SWV values of the two scanners were compared, significant differences between the devices were found in both muscles, orientations, and operators (see Tables 1 and 2 for exact *p*‐values). An exception was the BB muscle in the longitudinal transducer orientation in the measurements of the operator 2, in which no significant difference was seen between the two scanners (*p* = 0.109). The significance of the difference between the two scanners was stronger in the transverse orientation (*p* < 0.001 for both muscles regardless of the operator) than in the longitudinal (range of *p*‐values 0.019–0.109). Figure 2 demonstrates the difference of the two scanners and the codirectional measurements of the two operators.

In both muscles, the SWV values were overall slower in the transverse orientation of the transducer compared to the longitudinal orientation. The mean, minimum, and maximum SWV values and the SDs of the studied muscles are presented in Tables 1 and 2.

The SWV values were slower in the TA compared to the BB in the longitudinal orientation (*p* < 0.001 for measurements done by both operators and with both scanners). However, in the transverse orientation, a statistically significant difference was seen only in the measurements of the operator 2 done with the scanner 1 (*p* = 0.001).

**FIGURE 3:**
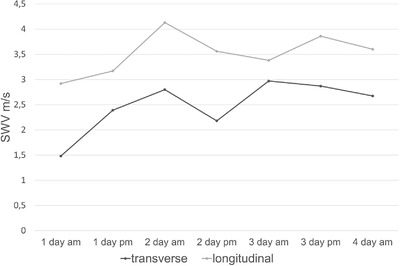
The graph demonstrates the steadiness of the shear wave velocity in the biceps brachii (BB) of one volunteer during a follow‐up period of 4 days within 1 week. The shear wave elastography was performed in the morning and afternoon. SWV, shear wave velocity; measure unit is m/s.

The graph demonstrating the SWV measurements over a 1‐week follow‐up period is shown in Figure 3. The transverse values ranged from 1.48 to 2.97 m/s. The longitudinal values ranged from 2.92 to 4.13 m/s. Considering that the measurements were performed with scanner 2, the SWV values are within the range of the minimum and maximum values of the larger material presented in Tables 1 and 2. The mean SWV values of both transverse and longitudinal measurements were within the first SD of the larger material.

## 4 DISCUSSION

The interobserver reliability between the two operators was very good with both scanners. However, the measurements of both operators showed significant differences between the scanners in the SWV values of both muscles and transducer orientations. The divergence of the scanners was emphasized in the transverse orientation in both muscles. According to these results, the reproducibility of the SWE seems to depend more on the used US device than on the operator. The good interrater agreement of SWE is in‐line with most previous studies.^17^, ^18^ It also indicates that SWE can be performed reliably even with lesser user experience, with previous hands‐on training and by committing to the previously proposed practical guidelines.^3^, ^19^ SWE also seems to be rather repeatable between days; however, this result of the current study should be considered only directional, as the study period and material size were very limited.

The presented good interrater agreement represents an optimal situation, where the SWE study protocol is agreed and highly standardized between the operators. In the present study, the operators were allowed to see the measurement events of each other but were blinded to the actual acquired SWV values. This allowed the study protocol to be replicated in detail. The manufacturers SWE default protocol was used in both scanners. The default protocols were not described in detail, and possible differences in the settings may explain the divergence of the scanners. There may also be differences in the speckle tracking algorithms between the scanners.

A widely acknowledged muscle SWE protocol for general clinical and research use has not yet been introduced, which limits the comparability of studies on SWE. For instance, SWE is reliant on the stretch properties of the muscle and consequently on the limb position.^13^ Stretching of the muscle is known to enhance the propagation velocity of the shear wave pulse, resulting in faster SWV values. Chen and coworkers demonstrated that stiffness in BB is significantly higher at full extension of the elbow compared to 30° flexion.^20^ In the study of Chen and coworkers, SWV in the BB was 2.62 ± 0.22 m/s in full extension and 2.26 ± 0.27 m/s at 30° flexion of the elbow with a longitudinal transducer orientation. Interestingly, in our study, the SWV in the BB was remarkably higher with both scanners compared to the results of Chen and coworkers, regardless of the same limb position (full extension of the elbow). It can be questioned whether there is after all a difference in the study methods, or could the discrepancy be explained by the different US devices. Moreover, in a clinical setting, it may be difficult to keep the tissue immobile and unstressed during the measurement.

The SWV is also dependent on the placement of the ROI.^3^, ^6^ In a recent study made with phantoms, Alrashed and Alfuraih demonstrated that the performance of the US devices to detect increasing stiffness is remarkably impaired in deeper targets.^21^ They also showed that different US devices could give more concordant results in shallower targets than in deeper (1.5 vs. 5 cm).^21^ However, in clinical use, a 1.5‐cm depth is not always optimal. Even though superficial muscles were chosen in our study, the depths of middle portions of the BB and TA muscles vary. Placing the ROI more superficially instead of the center of the muscle could result in a better reproducibility.

Study limitations of the present study include the relatively limited operator user experience of the SWE application, regarding the reliability of the interrater agreement. However, a systematic error in such scale, resulting in a consistent falsely good interrater reliability, can be considered unlikely. However, it would have been preferable for one of the operators to be more experienced than the other to study the experience dependency more accurately. This setting was performed in the study of Phan and coworkers, in which one operator with more than 30 years of expertise and a second operator with 1 year of expertise resulted in an ICC > 0.97 in the SWE of the BB muscle.^22^

Another limiting factor in the present study was that the distribution between the two sexes was uneven and age range was limited, as all participants were of working age, and the female gender was emphasized. SWE values have been shown to be dependent on age and sex,^23^ which should be noted in the clinical use of SWE. On the other hand, the effect of the variable patient characteristics to the study results can also be questioned. Additional repetition of the measurements in a phantom with known characteristics would have given a reference to the results obtained from the muscle tissue. However, the study aimed to evaluate the reproducibility of SWE particularly in a clinical context. It is also noteworthy that the lack of systematic muscle relaxation time before the SWE may have caused some measurement error. In addition, more repetitions could have been done to improve the reproducibility. Finally, the exact size of the ROI also affects the reproducibility; however, the ROI was standardized in our study to 3 mm.

We conclude that in further studies, a standardized protocol in repetition of the SWE should be agreed, because many adjustable variables, for instance, limb position and placement of the ROI, affect the SWE values and the reproducibility. The very good interrater agreement in our study shows that with a standardized study protocol, SWE can be reproduced reliably. However, the US device dependency cannot be excluded, and it may be preferable that clinics collect reference values with their own US device and consider threshold values presented in previous studies only directional.

## AUTHOR CONTRIBUTION

All of the authors contributed to the design and implementation of the research, to the analysis of the results, and to the writing of the manuscript.

## CONFLICT OF INTEREST

The authors declare that there is no conflict of interest that could be perceived as prejudicing the impartiality of the research reported.
